# Imaging of artificial tumor models in an anatomical breast phantom with a single-sided magnetic particle imaging scanner

**DOI:** 10.21203/rs.3.rs-6486706/v1

**Published:** 2025-05-20

**Authors:** Alexey Tonyushkin, Christopher McDonough, John Chrisekos, Matthew Jurj, Alycen Wiacek

## Abstract

Magnetic Particle Imaging (MPI) is an emerging biomedical imaging modality that detects superparamagnetic iron oxide (SPIO) nanoparticle tracers, providing high contrast, sensitivity, and quantification capabilities without the use of ionizing radiation, making it particularly suitable for cancer diagnostics. Considerable engineering efforts are underway to translate MPI technology to clinical settings. Most of these MPI scanners feature a cylindrical bore geometry similar to other clinical imaging modalities, which restricts their potential application primarily to head scanning. We have developed a single-sided MPI scanner designed to expand the technique's applicability to other regions of the human body. In this study, we demonstrate the imaging capabilities of our single-sided MPI scanner by imaging an anatomical breast phantom with concealed SPIO point sources to evaluate its potential for breast tumor diagnostics. Our method successfully distinguished these artificial tumors in two orthogonal slices, showcasing the scanner's capability to facilitate the transition of MPI technology from small animals to clinical use.

## 1 Introduction

Breast cancer is the most prevalent cancer among women worldwide and in the United States [1]. The current standard screening method, x-ray mammography, has an overall sensitivity of around 80% [2]. However, its effectiveness significantly decreases for women with dense breast tissue, leading to higher rates of false positives and false negatives [3]. Additionally, mammography is associated with considerable patient discomfort. Other imaging modalities, such as magnetic resonance imaging (MRI), ultrasound, computed tomography (CT), and single-photon emission computed tomography (SPECT) used for molecular breast imaging (MBI), are available but are generally used as supplemental techniques due to limitations in sensitivity, cost, imaging time, and the use of ionizing radiation [4]. These challenges highlight the need for a novel screening approach that is safe, highly sensitive, cost-effective, and robust.

In addition to early detection, precise localization of malignant cells is crucial for effective breast cancer treatment. Breast-conserving surgery remains a preferred option, but complete tumor removal is essential to prevent recurrence. Therefore, intra-operative imaging techniques to delineate tumor margins are currently under development [5]. One example of an emerging method to distinguish normal from cancerous tissue is photoacoustic imaging. This non-invasive, high contrast imaging method visualizes differences in optical absorption using a pulsed laser to elicit an acoustic response from tissue [6].

Magnetic Particle Imaging (MPI) is another emerging tracer-based biomedical imaging modality capable of detecting superparamagnetic iron oxide (SPIO) nanoparticles with exceptional sensitivity, contrast, and high spatiotemporal resolution [7]. MPI is considered a safe imaging technique, as it does not involve ionizing radiation and utilizes non-toxic SPIO tracers, some of which have been approved for use in magnetic resonance imaging (MRI) applications [8, 9]. Similarly to MBI, MPI has the potential to use specific biomarkers to distinguish between malignant and benign tumors by conjugating SPIO with a chemical agent [10]. Therefore, the MPI technique could provide a transformative alternative to current standards in both diagnostic imaging and tumor margin detection, improving diagnostic accuracy and patient outcomes.

Over the past decade, MPI technology has advanced rapidly, leading to the development of commercial preclinical MPI systems with small-bore designs, such as those offered by Bruker (Karlsruhe, Germany) and Magnetic Insight (Alameda, CA). The utility of MPI imaging was demonstrated in a range of preclinical applications such as angiography [11, 12] and perfusion [13]; cancer imaging [14], cell tracking [15, 16], hyperthermia [17, 18], and real-time tracking of interventional instruments: e.g., balloon catheters in cardiovascular procedures [19, 20]. Significant research efforts are underway globally to develop human-sized MPI systems, with a primary focus on closed-bore, head-only scanners [21–24] and targeted applications in functional MPI [25] and cancer detection and treatment [26]. Recently utility for interventional procedures was demonstrated with the leg interventional scanner, iMPI [27, 28]. Despite these advancements, the extension of MPI technology to whole-body imaging remains challenging due to safety concerns and the high power requirements associated with closed-bore geometries [29].

In this work, we explore the suitability of an MPI scanner with an asymmetric geometry for breast cancer diagnostics. A single-sided MPI scanner has all hardware on one side [30–32], allowing unrestricted access to the imaging subject. This topology enables imaging of small regions of interest in large animals or human subjects, making it a promising candidate for breast cancer imaging applications. Recent developments in SPIO detectors include handheld probes, which can be helpful in various breast cancer-related applications such as sentinel lymph node (SLN) biopsy [33–36] and breast-conserving surgery [37, 38]. These devices, however, are not capable of tomographic imaging and limit penetration depth due to low power, and thus excessive noise coupling and large magnetic field inhomogeneity. Consequently, a purely single-sided design is not a universal solution for imaging the whole body. However, integrating a magnetic field-free region in the form of a line referred to as a field-free line (FFL) gradient topology into a single-sided MPI scanner [39] could generate sufficient magnetic field gradients and excitation fields for imaging peripheral organs, including breast cancer screening, SLN imaging, and staging.

Our single-sided MPI scanner employs an FFL-based gradient field and a 25 kHz excitation field [40, 41], leveraging safety standards [42], high spatial resolution, and the enhanced sensitivity of FFL technology, which has been shown to provide a 10-fold gain in sensitivity over an alternative field-free point (FFP) configuration of field-free region while enabling robust image reconstruction [43]. Here, we demonstrated its feasibility for human breast imaging application by performing imaging experiments on an anatomical breast phantom exceeding the size constraints of commercial small-animal MPI systems. In our study, two spherical artificial tumor models filled with SPIO were embedded within the phantom. We conducted imaging in the coronal plane and 2D projection imaging in the axial plane, achieving spatial resolutions of 6 mm full-width at half-maximum (FWHM) in-plane, demonstrating sub-centimeter performance. These results were comparable to those achieved in MBI [44]. Thus validating our single-sided MPI scanner's ability to image larger objects beyond the capabilities of current commercial MPI systems while maintaining clinically relevant resolution.

## 2 Results

### 2.1 Single-Sided FFL MPI Scanner

Our single-sided FFL MPI scanner is designed to facilitate imaging of near-surface structures, such as breast tissue. Unlike conventional MPI scanners with closed-bore geometries, the single-sided design allows for imaging large or irregularly shaped objects. The system comprises three racetrack-shaped electromagnetic coils embedded in a custom-built water-cooled enclosure by Resonance Research Inc. (Billerica, MA, USA) as shown in Fig. 1(b,c), which generate the excitation and selection of magnetic fields required for imaging.

At the center of the scanner is the excitation coil, which produces an alternating magnetic field at a frequency of 25 kHz, referred to as the excitation field. This field is oriented along the z direction at the center of the FOV, and develops a small x component at lateral positions from the center. The direction of the excitation field is

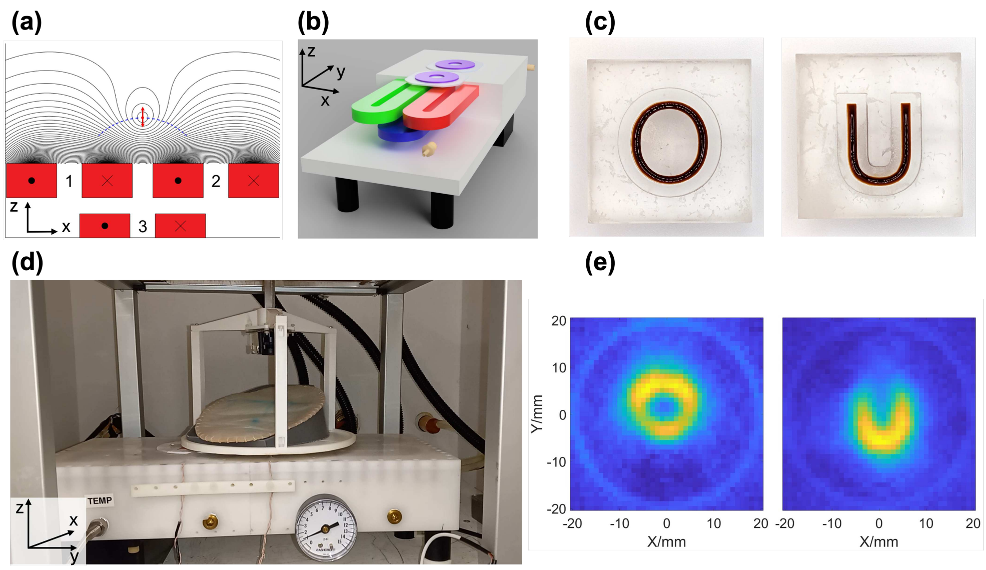

**Fig. 1 Setup of single-sided FFL scanner. a** Cross-sectional schematic of the single-sided MPI scanner, showing internal coils and magnetic field geometry. Selection coils (1 and 2, red blocks) are positioned on the scanner surface, with the excitation coil (3) located below. Black lines represent the static magnetic field generated by the selection coils, forming an FFL in the center of the scanner (red dot). The dashed blue arc indicates the range of FFL positions achieved by varying the relative currents. Red arrows show the direction of the excitation field. **b** 3D rendering of the scanner assembly. Selection coils (red and green) near the scanner surface, with excitation coil (blue) below them. The receive coil and gradiometer coils (purple) are located on the surface of the scanner. **c** Photograph of two SPIO-filled phantoms shaped as the letters "O" and "U". **d** Photograph of the experimental setup. A breast phantom is positioned inside a holder in the center of the sample table, which allows for rotation and vertical translation of the subject. **e** Examples of MPI images of "O" and "U" phantoms acquired with our single-sided scanner, demonstrating its ability to image complex geometries.

illustrated in Fig. 1(a) with red arrows. Nonlinear oscillations are induced in the SPIO by the excitation field and are detected by a circular receive coil located on the surface of the scanner. To suppress background signal originating from feedthrough with the excitation coil, an additional gradiometer circular coil, identical to the receive coil and functioning as a gradiometer, is positioned adjacent to it [45].

The selection coils are located beneath the surface of the scanner, equidistant from its center. These coils generate a gradient magnetic field that creates an FFL. This FFL represents the region of sensitivity for SPIO detection, as SPIO particles located outside this region produce negligible signals. Generating the FFL and scanning its position allows for spatial encoding of our signal. Due to the single-sided geometry of the selection coils, the trajectory of the FFL represents an arc shown in Fig. 1(a) as a blue dashed line. This trajectory can be linearized by implementing a linearization algorithm as suggested in [46].

The imaging subject is positioned on an 18.5 cm diameter sample table (see Fig. 1(c)) capable of rotation in the xy plane and vertical translation along the z-axis, allowing for mechanical depth encoding of the imaging volume. The imaging volume is located above the surface of the scanner, spanning a 4 cm × 4 cm plane located 17 mm above the device. Due to the single-sided nature of the scanner, the magnetic field strength varies with position. At the center of the scanner's surface, the excitation field amplitude is 1.2 mT. For coronal imaging, the gradient strength is maintained at 0.58 T/m across the imaging plane to ensure uniformity, which is necessary for avoiding artifacts when utilizing filtered back projection (FBP) reconstruction. In axial imaging, where FBP reconstruction is not required, a non-uniform gradient is used, achieving a field strength of 0.84 T/m at the center of the imaging volume.

### 2.2 MPI Imaging of Breast Phantom

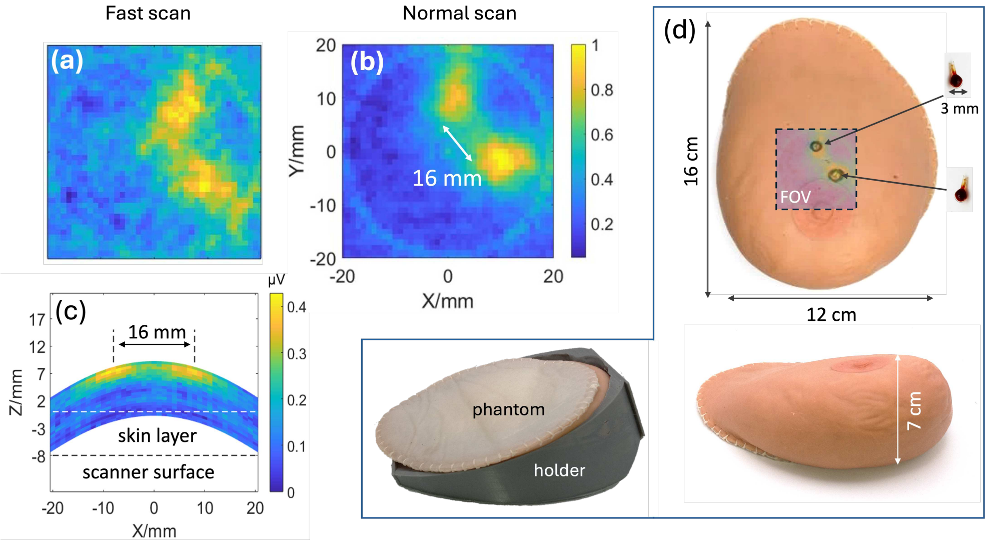

**Fig. 2 Anatomical breast phantom with MPI images. a** MPI image of artificial SPIO tumors from a fast scan (100 s) in a coronal plane. **b** High SNR MPI image in a coronal plane of tumors from a slow (10 min) scan. **c** MPI image of breast tumor model in axial plane (total acquisition time 10.5 min). **d** Anatomical breast phantom with labeled dimensions in two orthogonal views. Two 18 $\mu$l glass bulb SPIO (Synomag-D 50 nm) tumors are embedded in the breast phantom under the skin, placement shown as black circles, and the respective imaging FOV indicated as a square. Bottom-left: 3D printed holder for the breast phantom with the breast phantom placed inside in a prone position, used for consistent positioning of a phantom in the scanner.

To demonstrate the utility of the single-sided MPI scanner in accommodating the realistic human breast, we acquired an anatomical medical training breast phantom (SynDaver, FL, USA). While its complex tissue structure can be imaged with conventional imaging modalities such as ultrasound, MPI does not directly image the tissue. Therefore, artificial SPIO-labeled tumors were implanted into the phantom. Specifically, two small glass bulbs (18 $\mu$l each) were filled with undiluted Synomag-D 50 nm

(10 mg/ml) SPIOs to serve as artificial tumors. These “tumors” were implanted 7 mm beneath the skin of the phantom and positioned at the 12 and 1 o’clock locations, 39 mm and 20 mm from the nipple and spaced 16 mm apart. The breast phantom with the implanted tumors can be seen in Fig. 2(d). For imaging, the phantom was secured in a custom 3D-printed holder, oriented nipple-side down, and positioned on a rotation table above the scanner as shown in Fig. 1(d).

Two separate MPI imaging protocols were used to capture the complementary view of the SPIO tumors. The first protocol captured a 2D image in the coronal (XY) plane above the scanner. In this method, a pulse sequence was applied to acquire a 1D projection of the phantom, after which the phantom was mechanically rotated to capture projections at multiple angles. This process creates a sinogram that can be used to reconstruct a 2D image using FBP. To assess both clinical viability and scanner performance, two separate acquisition times were used. A short duration scan (100 s) was used to demonstrate that viable imaging can be performed under clinically practical time frames, and a longer scan (10 min) was performed to obtain a high signal-to-noise ratio (SNR) image.

In the second protocol, the phantom was imaged in the axial XZ plane to form a projection image. Similarly, a pulse sequence was applied to obtain a 1D projection, but instead of rotation, the phantom was mechanically raised 0.5 mm, and the field was electronically shifted to different points to sweep across the axial slice. This creates a 2D projection of the SPIO inside the imaged subject, with no reconstruction required. The total acquisition time for this sequence is 10.5 min.

The resulting images are shown in Fig. 2. The coronal image reveals two distinct hotspots corresponding to the tumor models, with a measured separation of 16 mm, consistent with their known implant location. The longer duration scan (Fig. 2(b)) provides high contrast images with clearly resolved tumors, while the shorter duration scan (Fig. 2(a)), though noisier, still allows both tumors to be distinctly resolved. The axial projection further confirms the lateral separation of the tumors and measures their depth of 7 mm below the surface of the skin. In both views, the tumors are clearly resolved with high contrast and no visible background signal from the surrounding tissue. This tracer-specific contrast represents a fundamental advantage of MPI over conventional modalities such as ultrasound, which generate a background signal from all tissue rather than targeted regions.

#### 2.3 Ultrasound Imaging of Breast Phantom

For reference to MPI imaging, ultrasound imaging was performed using a Clarius L7HD3 linear array (Clarius Mobile Health, Vancouver, Canada), and the position of the tumors were measured. The ultrasound scan revealed two tumors, with the bottom lateral edge of the transducer 1 cm from the nipple and in an approximately radial position. The resulting measurements from the ultrasound images confirmed that the artificial tumors were located 7 mm beneath the breast phantom’s skin and were separated by 16 mm, as shown in Fig. 3(b). Both MPI and ultrasound imaging successfully detected the SPIO-labeled tumors and allowed for the measurement of their relative positions within the phantom.

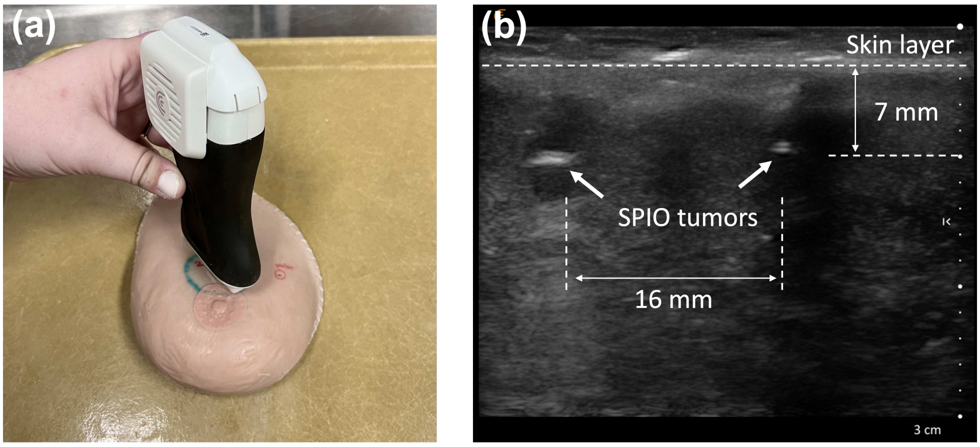

**Fig. 3 Ultrasound validation of SPIO tumor positions in anatomical breast phantom. a** Scanning with a handheld ultrasound imaging probe positioned on the top skin layer of the breast phantom. **b** Ultrasound image in axial plane showing two SPIO tumors located 7 mm beneath the phantom's surface, with a center-to-center separation of 16 mm. Dashed lines indicate the skin layer and horizontal positions of SPIO tumors.

Due to the small size of the SPIO tumor masses, they were difficult to visualize fully in the ultrasound images. However, the bright reflection off the glass bulb confirms the presence of the SPIO tumors. A dark artifact was observed beneath one of the tumors, likely caused by acoustic shadowing, which can occur when ultrasound waves are attenuated or reflected by dense or highly absorptive materials. Additionally, the inherent background signal from the phantom's tissue-mimicking material reduced the contrast between the SPIO tumors and the surrounding medium. While this limits the ability of ultrasound to directly visualize the tracer masses, it provides anatomical context that may be valuable when correlating ultrasound with MPI imaging.

### 2.4 Imaging Sensitivity: SPIO Dilution Experiment

To evaluate the imaging sensitivity of our MPI scanner, we imaged a series of dilution phantoms in addition to the breast phantom. In this study, the scanner has been upgraded with a new excitation coil to enhance field uniformity across the FOV. This coil provides a uniform 1.8 mT excitation field over the 4 cm FOV. The dilution phantoms consisted of the same 18 $\mu$l glass bulbs used as artificial tumors, but instead of containing undiluted Synomag-D 70 nm SPIO, they were filled with diluted SPIO solutions. Each glass bulb was positioned at the center of the imaging FOV, 17 mm above the scanner's surface.

Six dilutions were prepared at 1:1, 1:2, 1:4, 1:8, 1:16, 1:32 ratios. Given the initial Synomag-D concentration of 10 mg/ml and a bulb volume of 18 $\mu$l, the corresponding solid contents of SPIO were (180, 90, 45, 22.5, 11.3, 5.6) $\mu$g. The dilution phantoms are shown in Fig. 4(b). Imaging was performed with a 100 s acquisition time for all samples, except for the lowest concentrations (11.3 $\mu$g and 5.6 $\mu$g), which were averaged

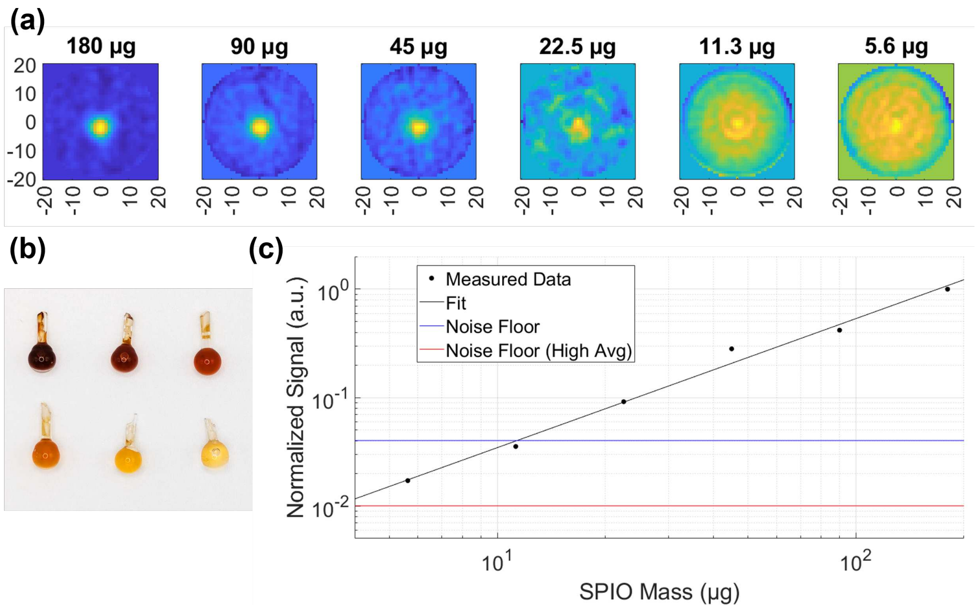

**Fig. 4 Sensitivity characterization using a dilution series of SPIO-filled glass bulbs. a** Coronal slices of MPI images of six glass bulb phantoms containing decreasing masses of SPIO (180 to 5.6 $\mu$g). **b** Photograph of the dilution series, showing the corresponding glass bulbs arranged by SPIO mass. **c** Signal plotted as a function of mass of solid SPIO content on a log-log scale. Measured values show a linear relationship between signal and iron content. The black line indicates the fit. Horizontal lines mark the noise floor (blue) and a lower noise floor for higher averaging conditions (red).

16 times, resulting in a total scan time of 27 minutes. Noise floor measurements were computed as the standard deviation of pixel values in regions without SPIO. Two separate noise floor values are reported, corresponding to the two separate acquisition times. If we assume particles consist of pure magnetite, then iron contributes to 72% of the total particle mass, and the lowest concentration would correspond to 4.0 $\mu$g of iron.

MPI images of the dilution phantoms and a plot of signal intensity versus dilution are presented in Fig. 4(a,c). This study demonstrates that our scanner can reliably detect iron masses as low as 4.0 $\mu$g with an SNR $> 1$.

## 3 Discussion

In this work, we successfully demonstrated a proof-of-principle MPI imager capable of imaging an anatomical breast phantom with lifelike properties and a largest dimension of 16 cm. Reconstructed FBP images showed two distinct point sources with well-resolved separation, though not representing the system's ultimate capability for spatial resolution [41]. Based on the point-spread function, the measured resolution in

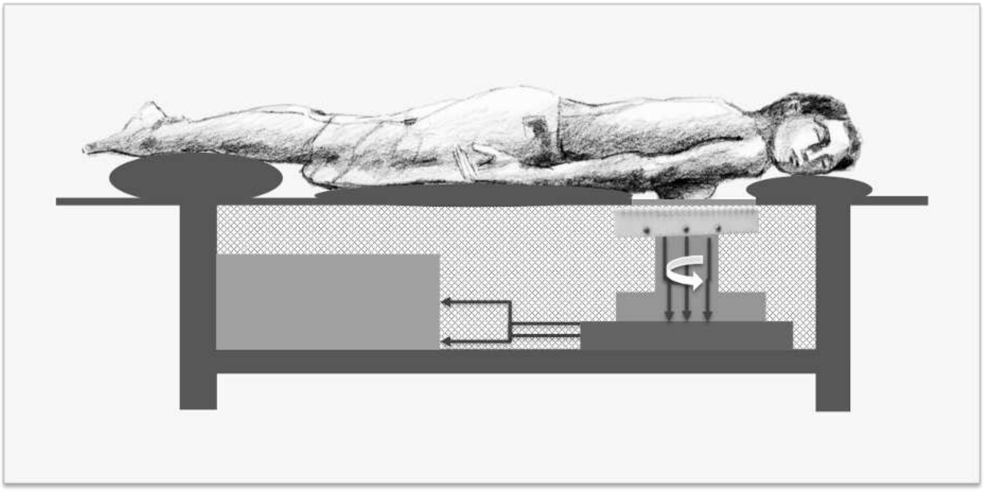

**Fig. 5 Conceptual design for a human-scale breast MPI scanner utilizing a single-sided FFL geometry.** An integrated single-sided MPI scanner with a patient table, showing the patient comfortably lying in the prone position.

the coronal plane at the available gradient strength was 6 mm FWHM, demonstrating sub-centimeter imaging performance.

For potential clinical translation to be used as a diagnostic breast imager, several key improvements have to be implemented as depicted in the sketch in Fig. 5 and are currently under investigation. First, the coil system has to be rotatable instead of the subject. Second, the imaging time of acquiring a slice has to be reduced to be within a minute. Third, the sensitivity has to be improved to allow less averaging time. Finally, the imaging depth should allow around 5 cm transversal FOV with sufficient gradient strength.

Previously, we demonstrated a sensitivity of our single-sided scanner up to 100 ng (Fe) in the MPI spectroscopy measurements using the same apparatus without image encoding [45]. To estimate the clinically relevant sensitivity, consider the average human injected dose (ID) of ∼400 mg (Fe) [47], the lower bound of a passive uptake of the SPIO by the tumor of ∼1 (%ID)/g [48, 49], then, for the average tumor mass of 1.2 mg per 1 $mm^3$ voxel [50, 51], the targeted sensitivity is 4.8 $\mu$g (Fe). Thus, the dilution imaging experiment (Sec.2.4) shows that we are currently capable of imaging clinically relevant SPIO concentrations. The future upgrades in coils and electronics would allow us to move closer to the ultimate 100 ng (Fe) sensitivity.

While MPI does not pose ionizing radiation risks, safety considerations are still necessary for human applications, primarily due to specific absorption rate (SAR) and magnetostimulation. These effects constrain the maximum excitation field amplitude in MPI. At frequencies below 42 kHz, magnetostimulation is the dominant safety concern, setting the excitation limit for full-torso imaging at 15 mT-pp for 25–50 kHz [42]. This threshold was estimated for full-torso imaging, and magnetostimulation limits scale inversely with object size $B_{lim} \propto \frac{1}{r_{obj}}$. Given an assumed breast radius of 80 mm (compared to a 20 cm torso radius), the magnetostimulation threshold for breast imaging is expected to be approximately 2.5 times higher, leading to an estimated limit of 37.5 mT-pp. Our scanner operates well within this limit, with an excitation field of 3.4 mT-pp, leaving room for increased sensitivity for future designs of our scanner.

The next generation of the scanner will be upgraded to implement a new breast-specific surface excitation coil and enhance the capability of selection field coils. This would allow us to increase the sensitivity of detecting the SPIO as well as to increase the FOV at least twofold while at the same time linearize the encoding trajectory. In addition, the upgraded device will be using a higher field gradient up to 1 T/m, which would allow increasing the spatial resolution to 4 mm in the coronal plane. As an alternative to all electromagnet design, the permanent magnet structure combined with electromagnets is shown to be efficient for the FFL single-sided scanner, which can provide 5 cm imaging depth with high gradients [52, 53].

Lastly, the robustness, imaging speed, and image quality can be improved with the implementation of Deep Learning (DL) image reconstruction algorithms, which we demonstrated through simulation [54]. Such DL imaging looks especially promising in the next iteration of the imager with a large FOV.

As the next step towards breast imaging applications, we plan to validate our technique with *in-vivo* imaging of breast tumor mouse models. Although cancer detection in rodent models was previously demonstrated using a small-bore rodent MPI scanner [14], to date, no *in-vivo* MPI imaging has been demonstrated utilizing a single-sided MPI scanner that can accommodate large subjects. We will consider a passive accumulation of SPIO in tumors through the enhanced permeability and retention (EPR) effect.

## 4 Materials and Methods

### 4.1 MPI Scanner Details

The single-sided MPI scanner consists of three racetrack-shaped electromagnetic coils embedded in a custom water-cooled enclosure. Two outer coils function as selection coils and are located 7 mm below the scanner surface. Located in the center of the scanner, below the two selection coils, is the excitation coil. All three coils were wound using stacked 0.82 mm $\times$ 2 mm rectangular copper conductor. Each selection coil is composed of eight stacks of racetrack-shaped layers (26 turns per layer), measuring 30.5 cm in length, 5.5 cm in width, and 1.6 cm in thickness, with an 11 mm gap between inner edges. The excitation coil is composed of six layers (also 26 turns each) with the same length, width, and a thickness of 1.2 cm. The selection and excitation coils have DC resistances of 140 m$\Omega$, and measured inductances of 360 $\mu$H and 349 $\mu$H at 25 kHz, respectively. The selection coils are powered independently by programmable DC supplies (Delta Elektronika SM6000 series), enabling electronic control of the FFL position across the imaging volume [41]. During scanning with a uniform gradient of 0.58 T/m, the average power dissipated in the selection coils is 0.89 kW.

The excitation field is driven at 25 kHz by a DS360 function generator (Stanford Research Systems), amplified by a Techron 7548 power amplifier (AE Techron), and

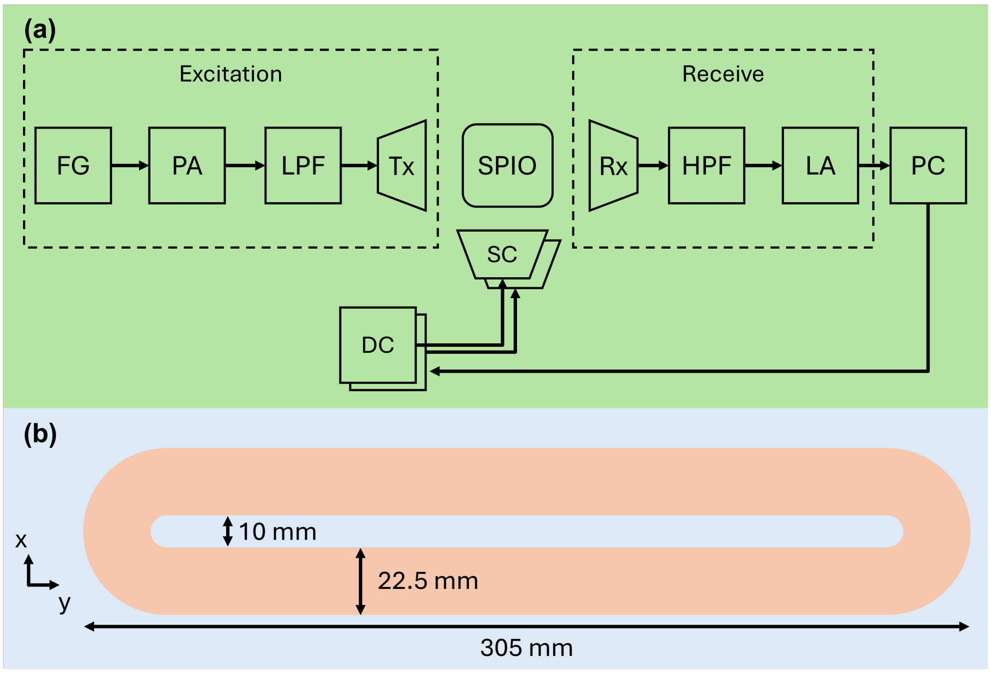

**Fig. 6** **Signal chain and coil geometry of single-sided MPI system. a** Block diagram of signal chain including transmit and receive chains. The transmit chain consists of a function generator (FG), power amplifier (PA), and a custom low-pass filter connected to the excitation coil (Tx). The receive chain comprises a receive coil (Rx), a high-pass filter (HPF), a lock-in amplifier (LA), with a digital connection to a personal computer (PC) for data acquisition, connected via GPIB. Additionally, the PC is used to control two DC switching power supplies (DC), each used to power a selection coil for electronic positioning of the FFL. **b** Schematic representation of the internal coil geometry. Both excitation and the selection coils are 305 mm long with a 10 mm inner spacing and a 22.5 mm track width.

passed through a custom low-pass filter with a cutoff near 25 kHz. The filter, composed of Celem capacitors ($C_1 = 4$ $\mu$F, $C_2 = 1$ $\mu$F) and custom wound air-core Litz wire inductors ($L_1 = 17$ $\mu$H, $L_2 = 64$ $\mu$H), provides attenuation of 60 dB of the third harmonic, ensuring a clean 25 kHz sinusoidal waveform. The resulting $\sim$6.8 $A_{peak}$ current generates an inhomogeneous excitation field, with an amplitude of 1.6 mT at the scanner surface and an amplitude of 0.78 mT at a height of 17 mm above the scanner, corresponding to the center of the FOV. An overview of the excitation and detection signal chain is shown in Fig. 6.

Signal detection is performed using a custom wound circular receive coil [45] consisting of 38 total turns of AWG 22 Litz wire (40/38), in two layers. The coil is wound on a 3D printed ABS frame with an inner diameter of 21 mm, and an outer diameter of 60 mm, with a total thickness of 4 mm (including base), and a measured inductance of 121 $\mu$H measured at 25 kHz. This coil is mounted on the surface of the scanner and is paired with an identical coil, which acts as a differential gradiometer, attenuating 41 dB of direct feedthrough. The differential signal is then routed to a high-pass filter with a 50 kHz cutoff, which attenuates the fundamental frequency. The filtered signal is then input to an SR860 lock-in amplifier (Stanford Research Systems), which demodulates the amplitude of the third harmonic, the dominant harmonic after the fundamental. The measured third harmonic amplitude is transferred to a PC via a GPIB interface for storage and subsequent image reconstruction.

### 4.2 Breast Phantom details

The breast phantom (SynDaver, FL, USA) has realistic synthetic human tissue that mimics the mechanical, thermal, and physicochemical properties of live tissue. It consists of skin, subcutaneous fat, bulk fat, a natural wear layer of dead skin at the surface, and three discrete layers (epidermis, dermis, and hypodermis) that move independently from one another. The synthetic human tissues are made from salt, water, and fiber. The phantom internal structure is visible by the clinical standard imaging modalities such as ultrasound, MRI, CT, and X-ray.

To enable stable and repeatable positioning of the breast phantom during imaging, a custom-made holder was fabricated to conform to the phantom's geometry. To construct this form, a 3D image of the breast phantom was acquired using the WIDAR 3D Scan & Edit app with Apple iPhone 13, capturing a full 360° view of the object. The resulting mesh was imported into Fusion 360 (Autodesk) and used to generate a conformal mold by subtracting the breast phantom volume from a cylindrical shell. Producing a negative cavity stabilizes the phantom's position throughout scanning.

The resulting holder design was exported as an STL file and fabricated with a filament 3D printer (QIDI I-Fast). The holder was printed with a base thickness of approximately 2 mm to maximize usable FOV in the vertical direction of our FOV, while still preserving mechanical stability. The thin-wall structure allowed for consistent positioning of the phantom within the scanner's FOV across multiple scans.

### 4.3 MPI Imaging Protocol

To acquire images with our MPI scanner, the FFL is scanned across the subject to generate a 1D projection of the SPIO content. The FFL is discretely stepped by applying different DC currents to the selection coils inside the scanner. Scanning covers a 4 cm range, centered at the scanner's midpoint. Due to the geomtery of the selection coils, the FFL follows a fixed arc above the the scanner surface, reaching a maximum height of 17 mm at the center and decreasing down to 10 mm at the edges of the FOV $\pm 20$ mm.

The process begins by forming the FFL at the leftmost position, -20 mm. At this initial position, a 203 ms delay ensures sufficient time for eddy currents to dissipate before signal acquisition. The third harmonic signal is then recorded for 70 ms. Subsequently, the FFL is incremented by 1 mm, followed by a shorter 47 ms stabilization delay, as smaller position changes require less settling time than the initial setup. The third harmonic is recorded again for 70 ms at each new position. This procedure is repeated for 41 FFL positions, spanning from -20 mm to 20 mm, with each complete scan of the 4 cm range taking 5 seconds. To improve the SNR of the 1D projection, the process is repeated for multiple scans. For imaging of the tumors, six averages were used per projection, except for the fast coronal imaging, resulting in a total acquisition time of 30 seconds per projection.

To perform tomographic imaging in the XY plane of our scanner, multiple 1D projections of the sample are acquired at 20 different angles, resulting in a total imaging time of 10 min. After each 1D projection is measured, the sample is mechanically rotated by 9° using a custom-made rotation table. The FFL is then scanned across the subject again to obtain a projection at the new angle. This process is repeated for 20 angles, spanning 0° to 180°, to construct a sinogram of the subject. The sinogram is subsequently reconstructed into a 2D slice of the subject using FBP with a Hann filter. To ensure uniform spatial encoding, which is required for FBP reconstruction, the gradient is 0.58 T/m at every FFL position in the FOV.

To obtain a 2D projection image of the subject in the XZ plane, the sample is not rotated but is instead mechanically raised using the sample table. Imaging begins with the table positioned closest to the scanner's surface, with its bottom 6 mm above the scanner's surface. A 1D projection of the subject is acquired at this initial height. The table is then raised in 0.5 mm increments, and a new 1D projection is measured at each height. This process is repeated for 21 heights, covering a 1 cm FOV in the z-direction, and requires a total imaging time of 10.5 minutes. The final 2D projection is a collection of 1D projections at different heights. This image needs no reconstruction and is analogous to an X-ray image, as it is not tomographic. Because this requires no reconstruction, spatial encoding does not require homogeneity. We take advantage of this by increasing the gradient to 0.84 T/m at the center of the FOV, where gradient generation is more power efficient.

#### 4.4 Ultrasound Imaging Protocol

The ultrasound imaging was performed using the Clarius L7HD3 linear array ultrasound transducer (Clarius Mobile Health, Vancouver, Canada) connected to an Apple iPad Air to acquire an axial image of the phantom. Prior to imaging, ultrasound gel was applied to the breast phantom and the ultrasound probe to ensure proper acoustic coupling, and a disposable latex cover was placed over the probe for sanitation. The probe was manually positioned on the breast phantom, as shown in Fig. 3(a). The probe was swept across the phantom, similar to a clinical breast ultrasound exam, until two tumors were visible in the image as shown in Fig. 3(b). With the Clarius mobile application, the ultrasound image was frozen and saved to the local device. The depth of the identified tumors from the skin surface and the distance between the two tumors were then measured from the saved ultrasound image and compared to the respective measurements from the MPI image.

### 5 Conclusion

In this work, we successfully imaged a breast phantom with implanted SPIO tumors using our prototype single-sided MPI scanner. The results demonstrate clear separation and detection of two tumors separated by 16 mm, which was confirmed by ultrasound, validating the potential of MPI for breast cancer diagnostics. MPI offers distinct advantages over other imaging modalities, such as MRI or CT, by providing zero background signal from surrounding tissue, resulting in unambiguous high-contrast images. This capability is crucial for detecting small or diffuse tumor masses that might be missed with other techniques. Our unique single-sided design enables it to image phantoms larger than those accommodated by conventional closed-bore MPI scanners, making the topology well-suited for clinical applications involving human organs.
